# Ginsenoside Rg5 Targets PRDX1 to Disrupt Redox Homeostasis and Induce Mitochondria-Dependent Apoptosis in Human Hepatocellular Carcinoma HepG2 Cells

**DOI:** 10.3390/molecules31030557

**Published:** 2026-02-05

**Authors:** Hai-Lun Ye, Ya-Ni Wang, Gang-Ao Li, Xing-Hui Jin, Guan-Ting Wu, Yang Li, Ying-Hua Jin

**Affiliations:** 1 Key Laboratory for Molecular Enzymology and Engineering of the Ministry of Education, School of Life Sciences, Jilin University, Changchun 130012, Chinaliyang915@jlu.edu.cn (Y.L.); 2 Ginseng Research Institute, Jilin University, Changchun 130021, China

**Keywords:** peroxiredoxin 1, ginsenoside Rg5, apoptosis, ROS, cancer therapy, synergistic therapy, DOX

## Abstract

Hepatocellular carcinoma (HCC) remains one of the leading causes of cancer-related mortality worldwide, with limited therapeutic options and poor clinical outcomes. Mounting evidence suggests that targeting cancer-specific metabolic and redox adaptations represents a promising therapeutic strategy. Peroxiredoxin 1 (PRDX1), a key antioxidant enzyme that is frequently overexpressed in HCC, enables tumor cells to neutralize excessive reactive oxygen species (ROS), thereby sustaining survival and conferring therapeutic resistance. In this study, using human hepatocellular carcinoma HepG2 cells as an in vitro model, we identify ginsenoside Rg5 (Rg5) as a previously unrecognized small-molecule inhibitor of PRDX1. Structural and functional analyses demonstrate that Rg5 directly binds to the Asn145 residue of PRDX1, effectively suppressing its peroxidase activity. Mechanistically, this inhibition disrupts ROS detoxification in HepG2 cells, leading to mitochondrial ROS accumulation, activation of the intrinsic apoptotic pathway, and consequent HepG2 cell death. Additionally, Rg5 not only suppresses HepG2 cell survival but also acts synergistically with doxorubicin, a first-line chemotherapeutic agent, to markedly enhance antitumor efficacy and potentially mitigate chemoresistance. Collectively, these findings suggest that PRDX1 inhibition may represent a broadly exploitable vulnerability in liver cancer and establish Rg5 as a promising candidate for developing targeted and combinatorial therapies against HCC.

## 1. Introduction

The dynamic balance between redox homeostasis and reactive oxygen species (ROS) plays a dual and complex role in tumor initiation, progression, and therapeutic response [1,2]. While ROS act as signaling molecules to promote proliferation and metastasis, their excessive accumulation induces lethal cytotoxicity [3,4]. To survive, cancer cells maintain “tolerable” ROS levels by fine-tuning antioxidant defenses against metabolic stress, oncogene activation, and harsh microenvironments [5,6]. The peroxiredoxin (PRDX) family is central to this adaptive process [7,8].

PRDX1 [9], a typical 2-Cys peroxidase and one of the most abundant PRDX members, possesses unique dual functions: it protects against oxidative damage by degrading hydrogen peroxide and simultaneously regulates downstream signaling pathways as a redox “sensor/mediator” [10,11,12,13]. Dysregulation of PRDX1 strongly contributes to tumor progression. Clinical and experimental evidence shows that PRDX1 is frequently upregulated in solid and hematological malignancies [14,15,16], where it drives proliferation, metastasis, stemness maintenance, and treatment resistance. Conversely, genetic or pharmacological inhibition of PRDX1 sensitizes cancer cells to oxidative stress, impairs DNA damage repair, and suppresses tumor growth [13,17], thereby highlighting PRDX1 as a promising therapeutic target for disrupting redox homeostasis in cancer.

Ginsenosides represent a potent class of compounds capable of regulating redox homeostasis [18,19,20]. Increasing evidence indicates that rare ginsenosides, such as Rg3 [20], Rh4 [21] and Rg5 [22], elevate ROS levels in various cancer models, thereby inducing cell death or autophagy. Although their ability to disrupt redox signaling has been demonstrated, the direct molecular targets responsible for ROS generation remain unidentified.

By combining phage display and cellular thermal shift assays, we identified Rg5 as the most potent PRDX1-binding molecule among the rare ginsenosides. Using bioinformatics, enzyme kinetics, and molecular biology approaches, we identified the binding site between Rg5 and PRDX1 and confirmed its inhibitory effect on enzymatic activity. Signal transduction assays, along with PRDX1 knockout and overexpression models, further established PRDX1 as a critical mediator of Rg5-induced apoptosis. Additionally, we explored its adjuvant potential with doxorubicin (DOX) and demonstrated its ability to enhance therapeutic efficacy.

Together, these results define the Rg5–PRDX1–ROS axis, highlight the potential of natural products in redox-targeted cancer therapy, and establish a mechanistic foundation for developing Rg5-based interventions against PRDX1-driven malignancies.

## 2. Results

### 2.1. Abnormally High PRDX1 Expression Correlates with Poor Prognosis in Liver Cancer

**Figure 1:**
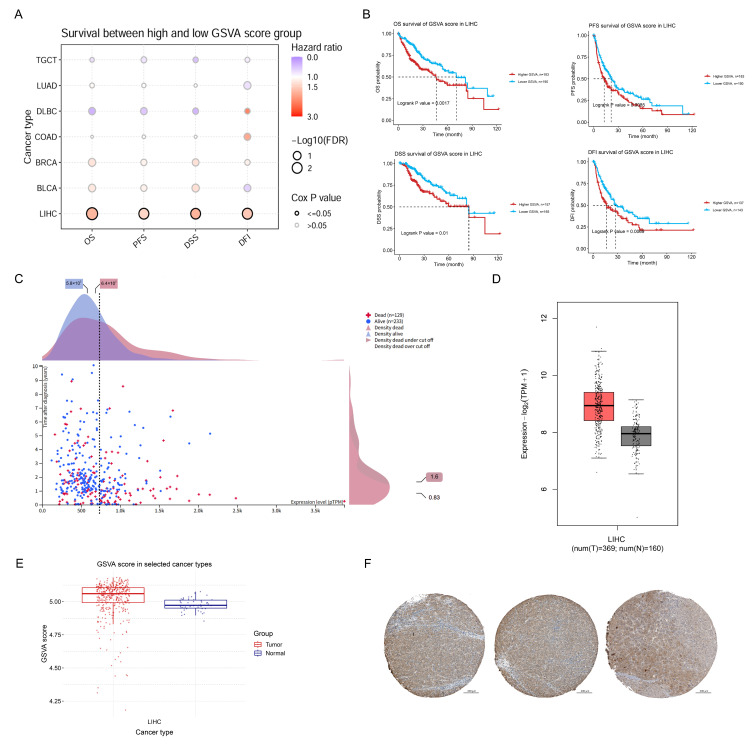
Elevated PRDX1 predicts poor survival in liver cancer. (**A**) Hazard scores of PRDX1 dysregulation in several common cancer types (from GSCA). (**B**) Impact of PRDX1 on four survival outcomes in liver cancer patients: overall survival (OS), progression-free survival (PFS), disease-specific survival (DSS), and disease-free interval (DFI) (from GSCA). (**C**) Scatter plot illustrating the association between PRDX1 expression and overall survival in HCC patients (from TCGA). (**D**) Transcriptional levels of PRDX1 in tumor versus adjacent normal tissues (from GEPAI-2). (**E**) Protein levels of PRDX1 in tumor versus adjacent normal tissues (from GSCA). (**F**) Immunohistochemical staining of PRDX1 in clinical HCC specimens (from THPA).

Based on our previous work, phage display screening identified PRDX1 as a potential binding partner of Rg5 (Figure S1). Pan-cancer analyses revealed that aberrant PRDX1 overexpression was associated with poor prognosis across multiple malignancies, with the strongest risk observed in HCC (Figure 1A). High PRDX1 expression correlated with reduced overall survival (OS), progression-free survival (PFS), disease-specific survival (DSS), and disease-free interval (DFI), confirming its strong prognostic impact (Figure 1B,C). Consistently, both transcriptomic and proteomic analyses on clinical big data (TGCA) demonstrated markedly elevated PRDX1 levels in HCC tumors compared with adjacent non-tumor tissues (Figure 1D,E), which was further validated by immunohistochemical staining showing high positivity rates in tumor samples (Figure 1F). Together, these findings establish aberrant PRDX1 expression as a key driver of HCC progression and a predictor of poor clinical outcome.

### 2.2. Rg5 Inhibits PRDX1 Enzymatic Activity Through Direct Interaction with the PRDX1-Asn-145 Site

**Figure 2:**
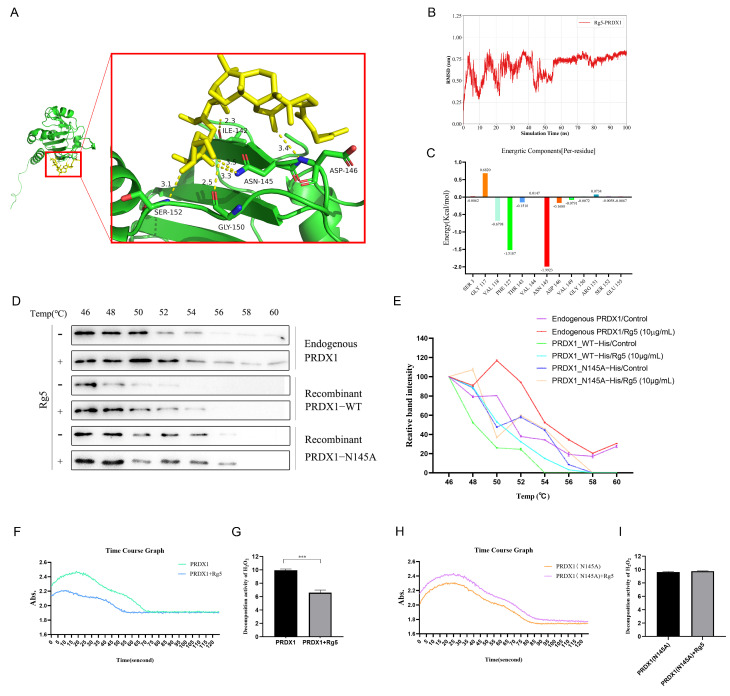
Ginsenoside Rg5 targets PRDX1 and inhibits its enzymatic activity. (**A**) Visualization of molecular docking between ginsenoside Rg5 and PRDX1. (**B**) Result of RMSD in molecular dynamics simulations between ginsenoside Rg5 and PRDX1. (**C**) Result of per-residue energy decomposition in molecular dynamics simulations between ginsenoside Rg5 and PRDX1. (**D**) Thermal shift assay of PRDX1 and PRDX1-N145A proteins following Rg5 treatment. (**E**) Thermal stability curves derived from thermal shift analysis. (**F**) Enzyme kinetic assay of PRDX1 based on H_2_O_2_ decomposition. (**G**) PRDX1 enzyme activity derived from enzyme kinetic analysis. (**H**) Enzyme kinetic assay of PRDX1-N145A based on H_2_O_2_ decomposition. (**I**) PRDX1-N145A enzyme activity derived from enzyme kinetic analysis. *** *p* < 0.001.

To validate the phage display results, molecular docking analysis was performed, revealing that Rg5 exhibited the lowest predicted binding energy with PRDX1 (binding score: −6.83) and interacted with five key residues: Asn145, Ile142, Asp146, Gly150, and Ser152 (Figure 2A). Molecular dynamics (MD) simulations further indicated that this binding conformation is likely to converge into a stable complex, with Asn145 contributing most significantly to the stabilization of the interaction (Figure 2B,C and Figure S3). To verify the critical role of Asn145, we generated the PRDX1-N145A mutant and conducted a protein thermal shift assay. Rg5 treatment markedly enhanced the thermal stability of endogenous PRDX1, increasing its melting temperature from 50 °C to 52 °C. A comparable stabilization effect was observed in purified recombinant PRDX1, with the melting temperature rising from 46 °C to 48 °C. In contrast, no detectable thermal stabilization was observed in the PRDX1-N145A mutant upon Rg5 treatment (Figure 2D,E).

Subsequently, we assessed the functional consequence of Rg5 binding using an H_2_O_2_ degradation assay. Rg5 treatment significantly inhibited the enzymatic activity of wild-type PRDX1, whereas the PRDX1-N145A mutant exhibited no appreciable change in activity (Figure 2F–I). Collectively, these findings demonstrate that Asn145 serves as a critical binding site for Rg5 on PRDX1 and that Rg5 effectively suppresses PRDX1 enzymatic function through direct interaction with this residue.

### 2.3. Downregulation of PRDX1 Induces Mitochondrial-Dependent Apoptosis

**Figure 3:**
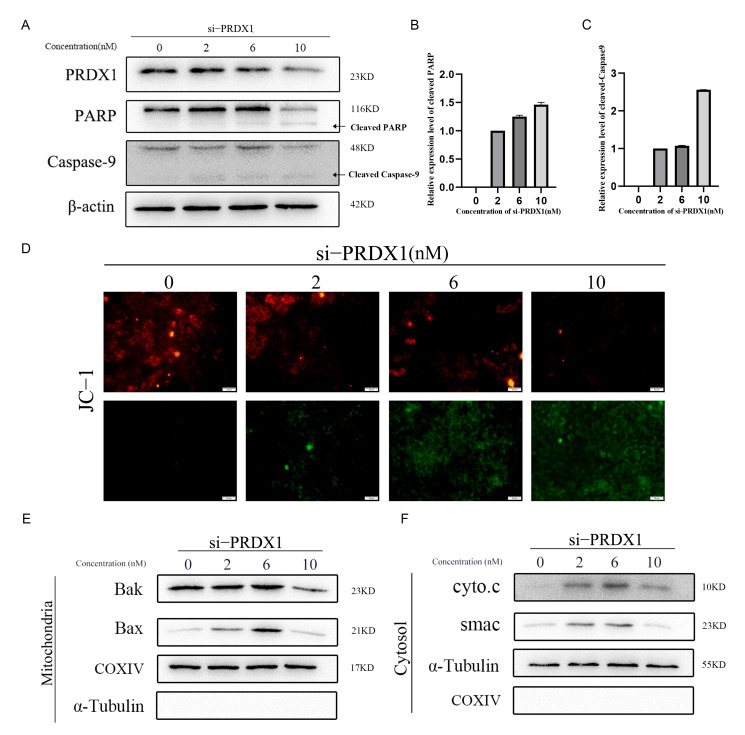
Low PRDX1 triggers mitochondrial apoptosis. (**A**) Expression and cleavage of apoptosis marker proteins PARP and Caspase-9 after PRDX1 knockdown. (**B**) Quantitative analysis of PARP cleavage band intensity following PRDX1 knockdown. (**C**) Quantitative analysis of Caspase-9 cleavage band intensity after PRDX1 knockdown. (**D**) Loss of mitochondrial membrane potential induced by PRDX1 knockdown. (**E**) Enrichment of Bak and Bax in mitochondria after PRDX1 knockdown. (**F**) Release of cytochrome c and SMAC into the cytosol upon PRDX1 knockdown.

To assess the essential role of PRDX1 on the hepatocellular carcinoma cell survival, we knock down it by its specific siRNA. Decreased PRDX1 expression led to significant cleavage of PARP, a substrate of caspase-3. At the same time, Caspase-9, the initiator caspase for the mitochondrial apoptosis pathway, was also observed to be activated (Figure 3A–C). JC-1 staining revealed a marked decrease in mitochondrial membrane potential following PRDX1 suppression (Figure 3D). Consistently, subcellular fractionation showed increased mitochondrial localization of Bax (Figure 3E), along with significant cytoplasmic release of cytochrome c and Smac (Figure 3F). Collectively, these results demonstrate that loss of PRDX1 compromises intracellular ROS clearance, thereby triggering severe apoptosis through the mitochondrial pathway.

### 2.4. Rg5 Targets PRDX1 to Trigger ROS Accumulation and Apoptosis

**Figure 4:**
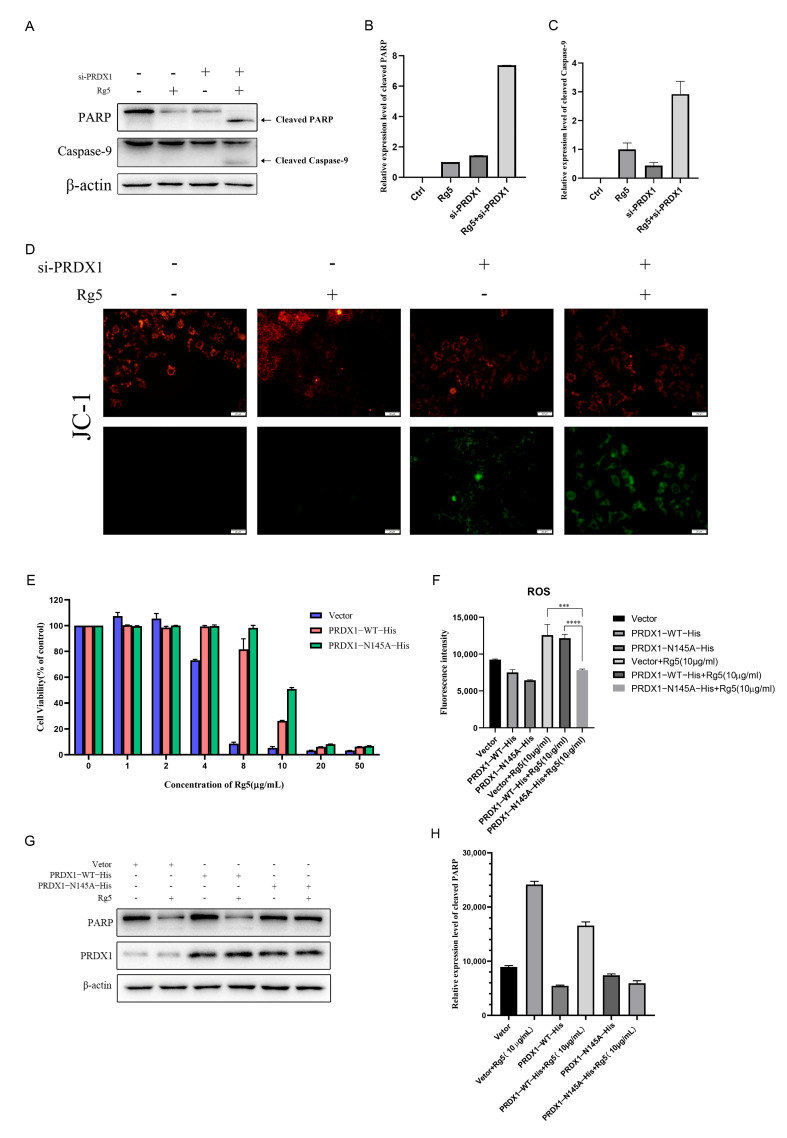
Ginsenoside Rg5 synergizes with PRDX1 knockdown to induce ROS-mediated apoptosis. (**A**) Expression and cleavage of apoptotic marker proteins PARP and Caspase-9 following co-treatment with Rg5 (5 μg/mL) and PRDX1 knockdown (si-PRDX1 10 nM). (**B**) Quantitative analysis of PARP cleavage bands after co-treatment. (**C**) Quantitative analysis of Caspase-9 cleavage bands after co-treatment. (**D**) Loss of mitochondrial membrane potential in cells after co-treatment with Rg5 (5 μg/mL) and PRDX1 silencing (si-PRDX1 10 nM). (**E**) Overexpression of PRDX1 and PRDX1-N145A confers resistance to Rg5-induced cytotoxicity. (**F**) Overexpression of PRDX1 and PRDX1-N145A significantly attenuates intracellular ROS accumulation. (**G**) Expression and cleavage of PARP visualized after overexpression of PRDX1 and PRDX1-N145A. (**H**) Quantitative analysis of PARP cleavage bands after co-treatment. *** *p* < 0.001, **** *p* < 0.0001.

After establishing the cytotoxic effects of Rg5 on hepatoma cells, we investigated whether this activity was mediated by PRDX1. Under optimized conditions, combined PRDX1 knockdown and Rg5 treatment significantly enhanced apoptosis, as evidenced by increased PARP cleavage and stronger caspase-9 activation (Figure 4A–C). This synergistic effect supports PRDX1 as a direct target for Rg5 in apoptosis induction. Further, the changes in mitochondrial membrane potential showed more prominent damage to the mitochondrial membrane potential after co-treatment (Figure 4D), which further provided a basis for Rg5 targeting PRDX1.

In contrast, PRDX1 overexpression conferred resistance to Rg5-induced cytotoxicity. Compared with vector controls, PRDX1 overexpression significantly improved HepG2 cell viability, with the PRDX1-N145A mutant showing stronger protective effects (Figure 4E). Consistently, PRDX1-N145A–overexpressing cells displayed reduced ROS accumulation (Figure 4F) and attenuated PARP cleavage (Figure 4G,H) compared with wild-type PRDX1-overexpressing cells. Collectively, these results demonstrate that Rg5 induces apoptosis in hepatocellular carcinoma cells by targeting PRDX1, causing excessive intracellular ROS accumulation and subsequent activation of the mitochondrial apoptotic pathway.

### 2.5. Rg5 Synergizes with DOX to Enhance ROS Accumulation and Apoptosis

**Figure 5:**
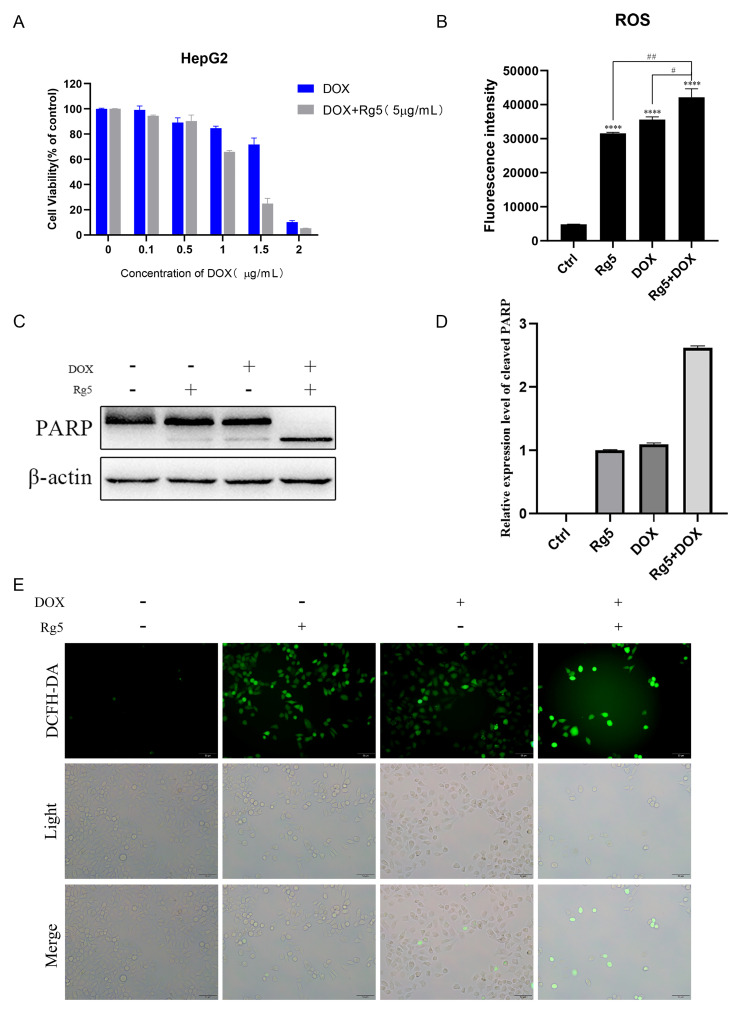
Ginsenoside Rg5 enhances DOX-induced apoptosis. (**A**) Cell viability assay of HepG2 cells treated with different concentrations of DOX (0.1, 0.5, 1, 1.5, 2 μg/mL) and DOX following Rg5 pretreatment (IC_50_ of DOX: 1.67 μg/mL; IC_50_ of DOX + Rg5: 1.05 μg/mL). (**B**) Enhanced accumulation of intracellular ROS after combined treatment with Rg5 and DOX. (**C**) Expression of PARP and cleavage bands after combined treatment with Rg5 (5 μg/mL) and DOX (1.5 μg/mL). (**D**) Quantification of PARP and cleavage bands after combined treatment with Rg5 and DOX. (**E**) Fluorescence labeling of intracellular ROS levels after combined treatment with Rg5 (5 μg/mL) and DOX (1.5 μg/mL). **** *p* < 0.0001 vs. ctrl. # *p* < 0.05, ## *p* < 0.01 vs. Rg5+DOX.

Furthermore, we examined the therapeutic synergy between Rg5 and doxorubicin (DOX), a clinically used ROS-inducing drug. Co-treatment with Rg5 significantly increased the cytotoxic effect of DOX (Figure 5A), accompanied by elevated intracellular ROS accumulation (Figure 5B). Consistently, PARP cleavage was markedly enhanced in the combination cells, indicating intensified apoptosis compared with either agent alone (Figure 5C,D).

Fluorescent ROS detection further revealed that although fewer cells remained viable after co-treatment, the ROS signal intensity per cell was substantially higher than in single-drug groups (Figure 5E). Collectively, these results demonstrate that Rg5 acts synergistically with DOX to amplify ROS accumulation and promote apoptosis in hepatocellular carcinoma cells.

## 3. Discussion

This study systematically demonstrated for the first time that the rare ginsenoside Rg5 directly binds to PRDX1, inhibits its enzymatic activity, and induces intracellular ROS accumulation, thereby activating the mitochondrial apoptosis pathway. Molecular docking, mutant protein validation, and enzyme kinetics analysis identified Asn145 as the critical amino acid residue mediating Rg5–PRDX1 binding. This interaction significantly suppressed PRDX1 enzymatic activity and initiated apoptosis via a mitochondrial-dependent mechanism.

Consistent with previous reports highlighting PRDX1 as a pro-survival antioxidant enzyme, our data further confirmed its central role in oxidative stress [9]. A knockdown study in HepG2 cells revealed that PRDX1 depletion disrupts mitochondrial homeostasis, as evidenced by decreased mitochondrial membrane potential, Bax translocation, and mitochondrial release of cytochrome c and Smac—hallmarks of mitochondrial apoptosis and Caspase-9 activation, as well as PARP cleavage. Furthermore, the combined treatment of Rg5 and PRDX1 knockdown demonstrated a synergistic pro-apoptotic effect. This was evidenced by enhanced cleavage of PARP and Caspase-9, along with a further decrease in mitochondrial membrane potential. Molecular dynamics simulations further indicated that Asn-145 plays the most critical role in maintaining the stable conformation of the Rg5-PRDX1 complex. Consistent with this, the PRDX1-N145A mutant protein exhibited a significantly reduced binding affinity for Rg5. Concurrently, overexpression of this mutant conferred substantial resistance to Rg5-induced cytotoxicity and markedly reduced the accumulation of reactive oxygen species (ROS). These findings collectively validate PRDX1 as a molecular target of Rg5 and underscore the importance of Asn145 in mediating the Rg5–PRDX1 interaction.

Importantly, this study also uncovered the potential of Rg5 as a sensitizer for doxorubicin (DOX), a first-line chemotherapeutic agent for liver cancer [23]. While DOX induces ROS accumulation, its efficacy is limited by drug resistance and systemic toxicity [24,25]. Our findings revealed that Rg5 significantly enhanced DOX-induced ROS generation and apoptosis, suggesting a synergistic therapeutic relationship. These results align with recent global research trends emphasizing the role of natural products as modulators of redox homeostasis and adjuvants to conventional chemotherapy, highlighting the translational potential of Rg5 in liver cancer treatment.

Nevertheless, this study has limitations. Although the present study was conducted exclusively in HepG2 cells, the mechanism identified may have broader relevance to hepatocellular carcinoma (HCC). PRDX1 is frequently overexpressed in HCC and plays a critical role in maintaining redox homeostasis, a process commonly exploited by tumor cells to support survival and therapeutic resistance. Given the central involvement of redox regulation and mitochondrial apoptotic pathways in HCC progression, inhibition of PRDX1 by ginsenoside Rg5 may represent a shared vulnerability in HCC cells with elevated antioxidant capacity. Nevertheless, HCC is a highly heterogeneous disease, and molecular differences among HCC cell lines may influence their sensitivity to PRDX1 targeting. Therefore, while the present findings provide mechanistic insight into the anti-tumor effects of Rg5 in HepG2 cells, the broader applicability of this mechanism to HCC should be regarded as exploratory, and further validation in additional HCC models will be required to define the therapeutic scope of Rg5. In addition, as PRDX1 is embedded within a broader antioxidant network involving other PRDX family members and glutathione peroxidases [26], the systemic interplay and compensatory mechanisms warrant further exploration. Finally, while we observed a synergistic effect between Rg5 and DOX, the precise pharmacokinetic and pharmacodynamic mechanisms remain incompletely understood and require comprehensive in vivo and clinical validation to establish therapeutic safety and efficacy.

With growing research on PRDX1, evidence suggests that in addition to its well-established peroxidase activity, PRDX1 also exerts important “non-enzymatic” functions. PRDX1 exerts critical non-enzymatic functions by acting as a molecular chaperone that regulates protein interaction networks, preserves proteostasis, and stabilizes the NRF2 pathway through interactions with ubiquitin ligase complexes [12], and it modulates key signaling mediators, including NF-κB [27], p53 [28], and ATM [29], thereby influencing cell proliferation, apoptosis, and genomic stability [30,31]. These non-enzymatic effects are critical for counteracting protein toxicity and maintaining cellular homeostasis. Although it remains unclear whether Rg5 binding to PRDX1 affects these non-enzymatic functions, current findings suggest the possibility that Rg5 may influence PRDX1-dependent regulatory networks, providing a potential avenue for constructing a signal regulation framework centered on Rg5–PRDX1 interaction and potential ideas for subsequent in-depth research.

In summary, this study provides novel mechanistic insights into how Rg5 directly binds to the Asn-145 site of PRDX1 to inhibit its enzymatic activity, leading to ROS accumulation and activation of mitochondrial apoptosis. Beyond its intrinsic anti-tumor effects, Rg5 also enhances the efficacy of doxorubicin, suggesting its potential as an adjuvant in chemotherapy regimens. Together, these findings not only broaden the understanding of PRDX1 in tumorigenesis and therapy resistance but also highlight the promise of natural products such as Rg5 as a foundation for developing next-generation redox-targeted anticancer strategies.

## 4. Materials and Methods

### 4.1. Cells and Plasmids

HepG2 (EallBio Life Sciences, Beijing, China, 06.0060) and Huh-7 (EallBio Life Sciences, 06.0059) were tested in Dulbecco’s modified Eagle Medium (Procell, Houston, TX, USA; PM150210) supplemented with 10% fetal bovine serum (FBS; Procell; C3010-0500) and 1% penicillin/streptomycin (Beyotime, Shanghai, China, C0222) and were cultured at 37 °C and 5% CO_2_. The expression vectors pEXS-DH and pcDNA3.1-His were used as the preservation vector in our laboratory. *E. coli*-competent cells Trans5α (CD201-01) and BL-21 (CD901-02) were purchased from Transgen (Beijing, China).

### 4.2. Ginsenoside Rg5

**Figure 6:**
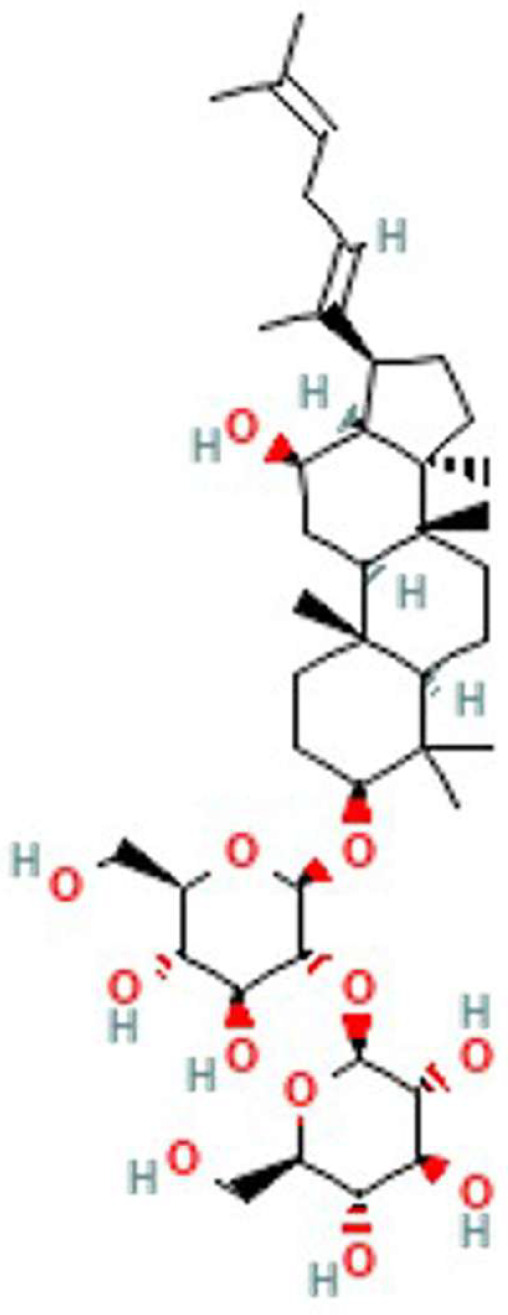
Molecular structure of Ginsenoside Rg5.

Ginsenoside Rg5 (Figure 6) with a purity ≥ 98% (V93778) was purchased from Shanghai Yuanye Company (Shanghai, China). Ginsenoside Rg5 was initially dissolved in 70% (*v*/*v*) ethanol to prepare a stock solution at a concentration of 10 mg/mL, which was aliquoted and stored at −80 °C until further use.

### 4.3. Clinical Data Analysis

Clinical performance analysis for PRDX1 is based on GEPIA2 (http://gepia2.cancer-pku.cn/#survival, accessed on 2 February 2026); GSCA: Gene Set Cancer Analysis (https://guolab.wchscu.cn/GSCA/#/expression, accessed on 2 February 2026); TGCA: The Cancer Genome Atlas Program (https://www.cancer.gov/ccg/research/genome-sequencing/tcga, accessed on 2 February 2026); THPA: The Human Protein Atlas (https://www.proteinatlas.org, accessed on 2 February 2026); and other clinical databases sharing data.

### 4.4. Molecular Docking

The molecular docking of ginsenoside Rg5 (Compound CID: 11550001) with PRDX1 (PDB ID: 7E4U) was performed using the AutoDock software (version 4.2.6). Prior to docking, all non-essential residues, water molecules, and heteroatoms were removed to define the active site of the target protein. The docking poses were visualized and analyzed using PyMOL (version 4.6.0), and the predicted binding sites were confirmed based on specific residue interactions.

### 4.5. Molecular Dynamics Simulations

Molecular dynamics (MD) simulations were performed using the GROMACS 2024 software package. The system was parameterized with the AMBER99SB force field, and the complex was solvated in a cubic box containing TIP3P water molecules. To maintain electrostatic neutrality, an appropriate number of sodium and chloride ions were added. Energy minimization was first carried out using the steepest descent algorithm to remove steric clashes. The system was then equilibrated through 100 ps simulations under constant volume and temperature (NVT) and constant pressure and temperature (NPT) ensembles, respectively. Subsequently, a 100 ns production MD simulation was conducted under periodic boundary conditions. Trajectory analyses were performed using built-in GROMACS utilities. The root-mean-square deviation (RMSD) of the protein backbone was calculated to assess structural stability, while the root-mean-square fluctuation (RMSF) was used to evaluate the flexibility of individual residues. The radius of gyration (Rg) was analyzed to characterize protein compactness, and the solvent-accessible surface area (SASA) was computed to describe protein–solvent interactions. In addition, free energy landscape (FEL) maps were constructed using RMSD and Rg values to identify conformational states. The binding free energy and per-residue energy decomposition were estimated from the equilibrated trajectories using the gmx_MMPBSA module, following the documentation provided at gmx_MMPBSA

### 4.6. Construction and Purification of PRDX1 Point Mutant Protein

The mutation design targeting Asn145 of PRDX1 was based on the gene sequence obtained from the NCBI database, and the resulting mutant sequence is provided in Supplementary Data Table S1. The designed mutation primers were cloned into the pEXS-DH expression plasmid. The recombinant plasmid was transformed into *E. coli* DH5α-competent cells for amplification, and the desired mutation was verified by DNA sequencing. The validated plasmid was subsequently transformed into *E. coli* BL21 (DE3) cells for protein expression. A single positive colony was inoculated into LB medium supplemented with 100 μg/mL ampicillin and cultured overnight at 37 °C with shaking. The overnight culture was diluted 1:100 into fresh medium and incubated until the OD600 reached 0.6–0.8. Protein expression was induced by adding 0.2 mM isopropyl β-D-1-thiogalactopyranoside (IPTG; MCE, Monmouth Junction, NJ, USA, HY-15921), followed by incubation at 16 °C for 16–18 h. After induction, the bacterial cells were harvested by centrifugation and lysed by ultrasonication in lysis buffer containing 1 mg/mL lysozyme (Sigma, Tokyo, Japan, 10837059001), 1 mM dithiothreitol (DTT; Sigma, D9779), and 1 mM phenylmethylsulfonyl fluoride (PMSF; Sigma, 282332). The lysate was centrifuged, and the supernatant was applied to a Ni–NTA affinity column pre-equilibrated with PBS buffer (Beyotime, C0221A). The bound proteins were washed and subsequently eluted with imidazole. The eluted fractions were concentrated and further purified using a 10 kDa molecular weight cutoff concentrator. The purified recombinant protein was concentrated, aliquoted, and stored at −80 °C for subsequent assays.

### 4.7. PRDX1 Enzyme Activity Assay

The enzyme activity of PRDX1 was determined by monitoring H_2_O_2_ degradation in the presence of ginsenoside Rg5. Purified Rg5 and PRDX1 proteins were mixed with 3% H_2_O_2_ solution at a molar ratio greater than 1:1 and incubated at 4 °C, protected from light, for 30 min. The enzyme kinetics parameters were subsequently recorded using a Shimadzu UV-2500 spectrophotometer (Shimadzu, Tokyo, Japan), and the data were analyzed according to standard procedures. One unit of PRDX1 enzymatic activity was defined as the amount of enzyme required to cause a decrease in absorbance of 0.01 per 10 s under the assay conditions.

### 4.8. Protein Thermal Shift Assay (PTSA)

Purified Rg5 and PRDX1 proteins were co-incubated at 4 °C for 1 h at a molar ratio of >1 (Rg5:PRDX1). The protein mixture was then aliquoted evenly into PCR tubes (100 μL per tube). Each aliquot was subjected to heating for 3 min at temperatures ranging from 44 °C to 62 °C in 2 °C increments (44, 46, 48, 50, 52, 54, 56, 58, 60, and 62 °C), followed immediately by incubation on ice for 5 min. Samples were subsequently centrifuged at 20,000× *g* for 15 min at 4 °C, and the resulting supernatant was collected for protein detection via Western blot analysis. For examination of endogenous protein interactions in cells, an excess of Rg5 was introduced to the cell suspension and allowed to co-incubate. After repeated freeze–thaw cycles, the supernatant was collected for further analysis.

### 4.9. Cell Proliferation Assay (MTT Assay)

Cells were seeded in 96-well plates at a density of 10,000 cells per well and allowed to adhere overnight. The cells were then treated with increasing concentrations of Rg5 (0–15 ng/mL) for 45 h. Following treatment, 20 μL of methylthiazolyl tetrazolium (MTT; 475989) solution (5 mg/mL) was added to each well, and the plates were incubated at 37 °C under 5% CO_2_ for 3 h. After incubation, the medium containing MTT was carefully aspirated. Subsequently, 150 μL of dimethyl sulfoxide (DMSO; 472301) was added to each well to dissolve the formazan crystals. The plates were gently agitated to ensure complete mixing, and the absorbance at 490 nm was measured using a microplate reader. All data were recorded and processed accordingly.

### 4.10. PRDX1 Gene-Silenced HepG2 Cell Construction

The siRNA for gene silencing was obtained from JTS Scientific (sequences are provided in Supplementary Data Table S2). HepG2 cells were plated in 6-well plates at a density of 100,000 cells per well and cultured overnight. For transfection, 5 μL of Lipofectamine 2000 (Thermo Fisher, Waltham, MA, USA; 11668030) and the appropriate amount of siRNA were each diluted in 250 μL of serum-free medium and incubated at room temperature for 5 min. The two solutions were then combined, mixed gently, and allowed to stand for 20 min at room temperature to form complexes. Meanwhile, the cell monolayers were washed and replaced with a small volume of serum-free medium. The siRNA-lipofectamine mixture was added dropwise to each well. Plates were gently rocked to ensure even distribution and incubated at 37 °C under 5% CO_2_.

### 4.11. Western Blot Assay

Cells were harvested using a cell scraper and collected by centrifugation with culture medium at 4 °C. The pellet was then resuspended and transferred, followed by lysis with an appropriate volume of RIPA lysis buffer (Sigma; R0278) for 55 min with gentle agitation at 4 °C to ensure complete lysis. After centrifugation at 12,000 rpm for 15 min at 4 °C, the supernatant was collected, and protein concentration was determined using the BCA protein assay for subsequent equalization. The samples were then boiled in loading buffer and subjected to SDS-PAGE electrophoresis. Following separation, proteins were transferred onto a PVDF membrane, which was subsequently blocked for 1 h at room temperature. The membranes were incubated overnight at 4 °C with the following primary antibodies: PRDX1 (CST, #8499, 1:3000), PARP (proteintech, 13371-1-AP, 1:2000), Caspase9 (proteintech, 10380-1-AP, 1:2000), β-actin (proteintech, 20536-1-AP, 1:2000), Bax (proteintech, 50599-2-Ig, 1:2000), Bak (proteintech, 29552-1-AP, 1:2000), COXIV (proteintech, 11242-1-AP, 1:2000), Smac (CST, #15108, 1:3000), cyto. *c* (proteintech, 10993-1-AP, 1:2000), and α-Tubulin (proteintech, 66031-1-Ig, 1:2000). After washing, membranes were incubated with a horseradish peroxidase-conjugated secondary antibody (CST #7074, 1:5000) for 1 h at room temperature. Protein bands were visualized using enhanced chemiluminescence (ECL) and quantified with ImageJ software (version 1.8.0). All experiments were performed in at least three independent replicates.

### 4.12. JC-1 Assay

HepG2 cells were seeded in 6-well plates at a density of 100,000 cells per well and cultured overnight. Following treatment, the original culture medium was aspirated and the cells were washed once with phosphate-buffered saline (PBS). Subsequently, 1 mL of serum-free medium was added to each well, followed by the addition of 1 mL of JC-1 staining solution. Cells were incubated with the JC-1 solution at 37 °C for 20 min in the dark. After incubation, the staining solution was removed, and the cells were washed twice with JC-1 staining buffer (1×). Fluorescence was then visualized by fluorescence microscopy or analyzed by flow cytometry. The Mitochondrial Membrane Potential Detection Kit (JC-1) was purchased from Beyotime Biotechnology (product code: C2006).

### 4.13. ROS Assay

HepG2 cells were seeded in 6-well plates at a density of 100,000 cells per well and cultured overnight. Following the indicated treatments, cells were harvested, collected by centrifugation, and resuspended. The cell concentration was adjusted to 5 × 10^6^ cells/mL in serum-free medium. The fluorescent probe 2′,7′-dichlorodihydrofluorescein diacetate (DCFH-DA) was diluted according to the manufacturer’s instructions and added to the cell suspension at a final concentration of 10 μmol/L. The mixture was incubated at 37 °C for 20 min in the dark, with gentle inversion every 5 min to ensure uniform exposure of the probe to the cells. After incubation, cells were washed three times with phosphate-buffered saline (PBS) to remove extracellular dye. Fluorescence intensity, corresponding to intracellular reactive oxygen species (ROS) levels, was measured using a fluorescence microplate reader. The Reactive Oxygen Species Assay Kit was obtained from Beyotime Biotechnology (product code: S0033S).

### 4.14. Statistical Analysis

Experimental data were obtained from independent triple-replicate experiments and were expressed as mean ± standard deviation (mean ± SD). GraphPad 9.0 software (GraphPad Prism, San Diego, CA, USA) was used for statistical analysis, Student *t*-test statistical analysis was used for comparison between groups, and *p* < 0.5 indicated that the difference between groups was statistically significant

## 5. Conclusions

This study provides the first systematic elucidation of the molecular mechanism underlying the rare ginsenoside Rg5 as a small-molecule PRDX1 inhibitor and its antitumor effects in hepatocellular carcinoma (HCC). Our results demonstrate that Rg5 directly binds to the critical residue Asn145 of PRDX1, efficiently suppressing its peroxidase activity and thereby disrupting intracellular ROS homeostasis. Molecular docking, mutant protein validation, and enzymatic kinetic analyses further confirmed the central role of Asn145 in the Rg5–PRDX1 interaction. Functional assays revealed that Rg5 markedly induces mitochondrial ROS accumulation and activates the intrinsic apoptotic pathway in HCC cells. In addition, Rg5 exhibits synergistic antitumor effects when combined with the frontline chemotherapeutic agent doxorubicin, highlighting its potential to enhance chemotherapy efficacy and overcome drug resistance.

In summary, this study not only unveils the druggable vulnerability of PRDX1 in HCC but also establishes Rg5 as a promising candidate targeting PRDX1, providing a novel theoretical basis for redox-based therapeutic strategies against liver cancer.

## Data Availability

The original contributions presented in this study are included in the article/Supplementary Materials. Further inquiries can be directed to the corresponding author.

## Supplementary Materials

The following supporting information can be downloaded at: https://www.mdpi.com/article/10.3390/molecules31030557/s1, Figure S1. Forty-six potential targets of ginsenoside Rh2 identified through phage display technology, along with ginsenosides screened based on structural similarity. Figure S2. Alterations in the Expression of Apoptosis-Associated Anti-Apoptotic Proteins Following PRDX1 Silencing. Figure S3. Molecular dynamics simulations. Table S1. Primer Design for Site-Directed Mutagenesis of PRDX1-N145A. Table S2. The si-RNA sequence for silencing the PRDX1 gene.
