# Supplementary material for: Ginsenoside Rg5 Targets PRDX1 to Disrupt Redox Homeostasis and Induce Mitochondria-Dependent Apoptosis in Human Hepatocellular Carcinoma HepG2 Cells
Source: Molecules. 2026 Feb 5;31(3):557. doi: 10.3390/molecules31030557 (PMC12899158; doi:10.3390/molecules31030557)
Supplement: Supplementary file 1 [file molecules-31-00557-s001.zip › molecules-4116293-supplementary.pdf]

Ginsenoside Rg5 Targets PRDX1 to Disrupt Redox Homeostasis and Induce Mitochondria-Dependent Apoptosis in Human Hepatocellular Carcinoma HepG2 Cells

Supplementary Figure

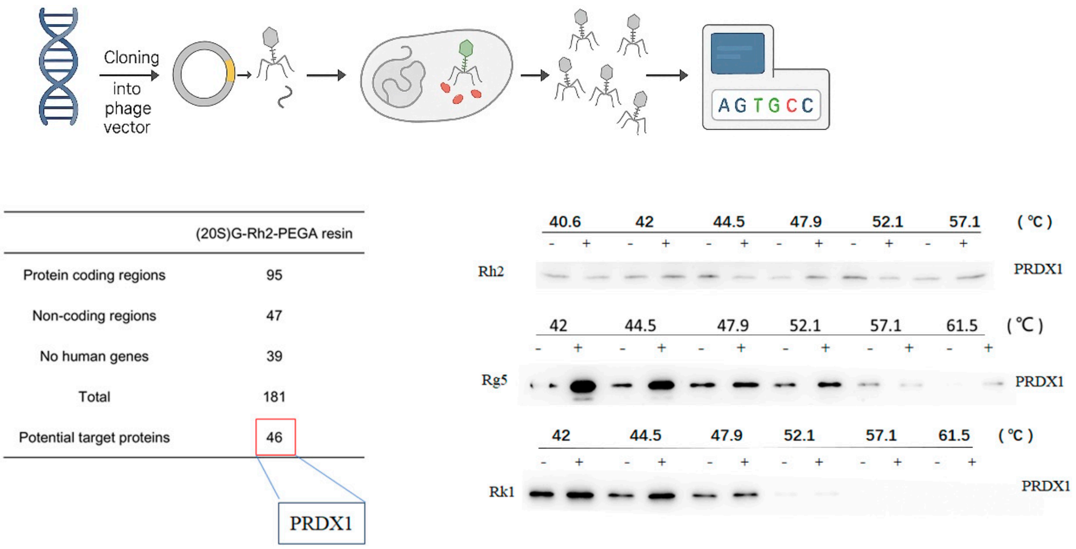

Supplementary Fig. S1 **Forty-six potential targets of ginsenoside Rh2 identified through phage display technology, along with ginsenosides screened based on structural similarity.**

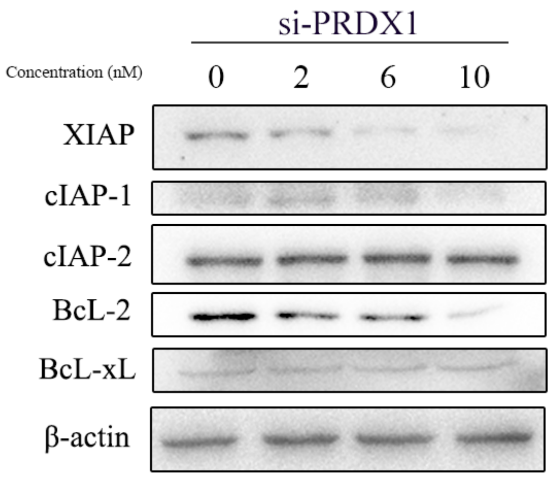

Supplementary Fig. S2 **Alterations in the Expression of Apoptosis-Associated Anti-Apoptotic Proteins Following PRDX1 Silencing**

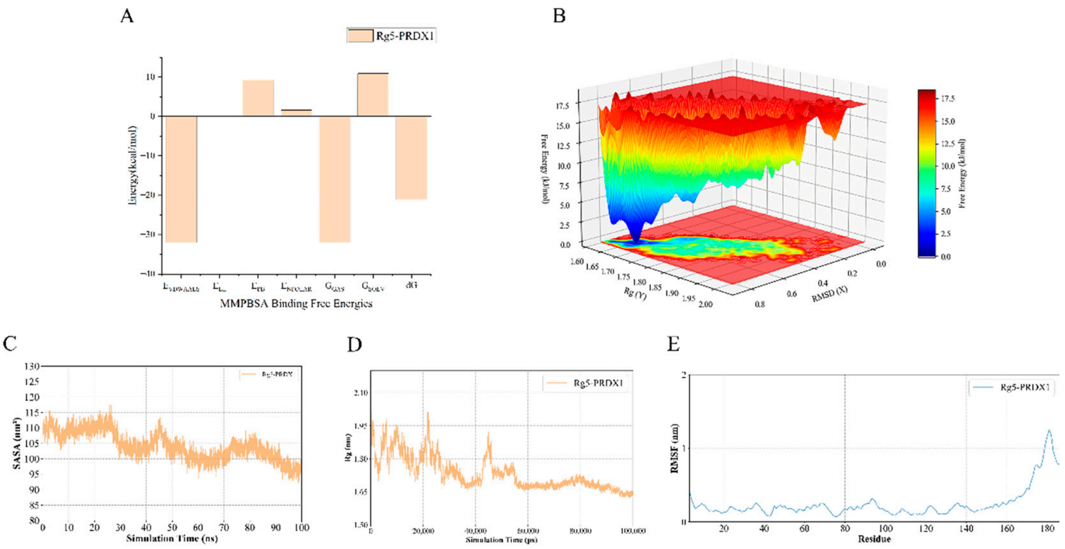

Supplementary Fig. S3 Molecular dynamics simulations

(A) Binding free energy decomposition analysis(MMPBSA) of Rg5 – PRDX1; (B)Three-dimensional free energy landscape of Rg5 binding to PRDX1; (C)Solvent-accessible surface area analysis(SASA) of the Rg5–PRDX1 complex; (D)Radius of gyration (Rg) analysis of the Rg5–PRDX1 complex; (E)Root mean square fluctuation (RMSF) analysis of the Rg5–PRDX1 complex.

Supplementary Table

Table S1. Primer Design for Site-Directed Mutagenesis of PRDX1 -N145A.

PRDX1-N145A	Primer sequence
Forward:	CGGCAGATCACTGTAGCTGACCTCCCTGTTG
Reverse:	CACAGAGCGGCCAACAGGGAGGTCAGCTACAG

Table S2. The si-RNA sequence for silencing the PRDX1 gene.

Si-PRDX1	Sequence
	GGUCAAUACACCUAAGAAATT<br>UUUCUUAGGUGUAUUGACVTT
